# FAIR Health Informatics: A Health Informatics Framework for Verifiable and Explainable Data Analysis

**DOI:** 10.3390/healthcare11121713

**Published:** 2023-06-11

**Authors:** Muhammad Hameed Siddiqi, Muhammad Idris, Madallah Alruwaili

**Affiliations:** 1 College of Computer and Information Sciences, Jouf University, Sakaka 73211, Saudi Arabia; 2 Universite Libre de Bruxelles, 1070 Brussels, Belgium

**Keywords:** data correlation, data linking, verifiable data, data analysis, explainable decisions, clinical trials, COVID, clinical investigation, semantic mapping, smart health

## Abstract

The recent COVID-19 pandemic has hit humanity very hard in ways rarely observed before. In this digitally connected world, the health informatics and investigation domains (both public and private) lack a robust framework to enable rapid investigation and cures. Since the data in the healthcare domain are highly confidential, any framework in the healthcare domain must work on real data, be verifiable, and support reproducibility for evidence purposes. In this paper, we propose a health informatics framework that supports data acquisition from various sources in real-time, correlates these data from various sources among each other and to the domain-specific terminologies, and supports querying and analyses. Various sources include sensory data from wearable sensors, clinical investigation (for trials and devices) data from private/public agencies, personnel health records, academic publications in the healthcare domain, and semantic information such as clinical ontologies and the Medical Subject Heading ontology. The linking and correlation of various sources include mapping personnel wearable data to health records, clinical oncology terms to clinical trials, and so on. The framework is designed such that the data are Findable, Accessible, Interoperable, and Reusable with proper Identity and Access Mechanisms. This practically means to tracing and linking each step in the data management lifecycle through discovery, ease of access and exchange, and data reuse. We present a practical use case to correlate a variety of aspects of data relating to a certain medical subject heading from the Medical Subject Headings ontology and academic publications with clinical investigation data. The proposed architecture supports streaming data acquisition and servicing and processing changes throughout the lifecycle of the data management. This is necessary in certain events, such as when the status of a certain clinical or other health-related investigation needs to be updated. In such cases, it is required to track and view the outline of those events for the analysis and traceability of the clinical investigation and to define interventions if necessary.

## 1. Introduction

Pandemics are not new to this world or humanity. There have been pandemics in the past, and they may happen again in the future. The recent COVID-19 pandemic is different from the previous ones in that the virus is more infectious without being known, symptoms are ambiguous, and the detection methods require a lot of time and resources. It has caused more deaths than ever in the history of humankind, and the impact it has had on the world economy and human lives (whether affected or not) is grave and is posing questions about the future of diseases and pandemics. While humans advance knowledge and technology, there is a need to investigate and put effort into overcoming the challenges posed by these kinds of serious threats. This does not only require us to deal with the current pandemic but also to look into the future, predict and presume the possibilities, investigate, and develop solutions at a large scale so that there is a reduction in the risk of losing lives and danger on a large scale.

The majority of the existing systems and solutions in the healthcare domain are proprietary and limited in their capacity to a specific domain, such as only processing clinical trial data without integrating state-of-the-art investigation and wearable sensor data. Hence, they lack the ability to present a scalable analytical and technical solution and have limitations and lack the ability to trace back the analysis and results to the origin of the data. Because of these limitations, any robust and practical solution does not only have to account for clinical data but also present a practical and broader overview of these catastrophic events from clinical investigations, their results and treatments that are up-to-date, and combine them with medical history records as well as academic and other related datasets available. In other words, the recent advancements and investigations in the clinical domain specific to a particular disease are published in research articles and journals, and they also need to be correlated to real human subjects that undergo clinical trials so that up-to-date analysis can be carried out and proper guidelines and interventions can be suggested.

Moreover, the majority of the record-keeping bodies maintain electronic health records for patients and, recently, records of COVID vaccinations. However, they lack the ability to link the data to individuals’ activities and clinical outcomes. There is also a lack of fusing data related to the investigation of a particular disease from various data providers, such as private trials, public trials [1,2,3], and public-private trials. The lack of these services is not only because there is less literature on fusing these multiple forms of data but also because of security and privacy concerns related to the confidentiality of healthcare data. The current advanced and robust privacy and security infrastructure available is more than enough to ensure personal and organizational interests. On the one hand, there are governments and other organizations that publish their clinical research data to public repositories to be available for clinical research using defined standards. On the other hand, there are pharma companies, which mostly hold the analytics driven by these and the respective algorithms and methods private. Furthermore, clinical trial data are not enough since they only provide measurements for different subjects who underwent a trial, while other data, such as electronic health records, contain real investigative cases and the histories of patients. Therefore, fusing these data from variety of sources is helpful to determine the effects of a particular drug/s or treatment plan in combination with other vaccines, treatments, etc.

Given the above brief overview of the capabilities of the state-of-the-art, most of these systems either tackle static or dynamic, relational or non-relational, noisy or cleaned data without fusing, integration, or semantically linking. This paper proposes a solution to the above problems by presenting a healthcare framework that supports the ability to acquire, manage, and process static and dynamic (real-time) data. Our proposed framework is a data format that focuses on fusing information from various sources. In a nutshell, the proposed framework ideally targets the strategy FAIR [4]: F: Findable, A: Accessible, I: Interoperable/Interchangeable, and R: Reusable.

### Objectives and Contributions

In particular, we define the objectives and contributions of this research work as follows: Propose a framework that is able to provide the following basic and essential capabilities for health-care data:

- Design of a clinical `data lakehouse` that stores data in a unified format where the raw data can be in any format loaded from a raw storage or streamed.
- Design of pipelines that support static and incremental data collection from raw storage to a clinical data lakehouse and maintain the trace of any change to the structure at the data level and at the schema level all the way to the raw data and raw data schema.
- Design of a schema repository that versions the data when it changes its structure and enables forward and backward compatibility of the data throughout.
- Design of universal clinical schemas that can incorporate any clinically related concepts (such as clinical trials from any provider) and support flexibility.
- Design processes to perform change data capture (CDC) as the data proceed down the pipeline towards the applications, i.e., develop incremental algorithms and methods to enable incremental processing of incoming data and keep a log of only the data of interest.
- Build timelines for changing data for a specific clinical investigation from a clinical trial data element such as Trial, Site, and Investigator, etc., and include the ability to stream the data to the application.
- Provide for semantic linking and profiling of subjects in the data.

These capabilities are meant to provide evidence of data analysis (i.e., where does the analysis go back in terms of data), traces of changes, and a holistic view of various clinical investigations running simultaneously at different places related to a certain specific clinical investigation. An example of that is when COVID-19 vaccination was being developed; it was necessary to be able to trace the investigation of various efforts by independent bodies in a single place where one could see the phases of clinical trials of vaccines, their outcomes, treatments, and even the investigation sites and investigators. This could not only result in better decision-making but also in putting resources in suitable places and better planning for future similar scenarios.

## 2. State of the Art

Many works exist in the literature that deal with various aspects of clinical data, ranging from data management and analysis to interoperability, the meaning of big data in healthcare and its future course, the standardization of clinical data, especially clinical trials, and the correlation and analysis of data from various sources. The following sections present a brief overview of existing works in these various aspects.

### 2.1. Data Management

Much work exists in data management in the healthcare domain. These include the design and analysis of clinical trials [5], big data in healthcare [6], and others [7]. A detailed survey in [8] can be seen by the readers for an overview of big data in health care. The crux of all the work in data management is to store data on a large scale and then be able to process it efficiently and quickly. However, this is not the only scope of this paper, since this paper does not only communicate and present information about data management (where we resemble the existing work) but also presents additional key features that distinguish our work from the existing work. Those distinguishing features are presented as contributions in the introduction section.

### 2.2. Data Interoperability and Standardization

Data interoperability and standardization are two other key aspects of healthcare data management and analysis. Unlike other traditional data management systems, healthcare data, in particular clinical data, require more robust, universally known, and recognized inter-operable methods and standards because the data are critical to healthcare and the healthcare investigations and diagnoses that come with it. For example, HL7 standards focus on standardizing clinical trials terminologies across various stakeholders in the world. Multiple investigation research centers need to exchange results, outcomes, treatments, etc. to come to a common conclusion for certain diseases and treatments. Therefore, a high quality of standards, as explained in [9,10], exists, and ontological [11] representations have been defined to represent the data in an interoperable manner universally. There are a wide range of resources in this domain, and the readers can see further details in the survey at [12]. Our research scope goes beyond this topic of standardization and interoperability and focuses on a more abstract level where all data from all types of providers in all standards can be brought together for analysis and be able to incorporate changes and evolve as the timelines of investigations evolve.

### 2.3. Data Analysis

Like any other field of data analysis, clinical healthcare data has also been widely studied for analysis and preparation. Such as the strategies for dealing with missing data [9], correlating data from various sources and generating recommendations for better healthcare [13], and the use of machine learning approaches for investigating the diagnosis of vaccines, e.g., COVID-19 vaccines [14]. Some examples of works related to this specific field are presented in [12,15]. Moreover, we also find efforts that investigate supporting clinical decisions by clinicians in various fields, and these types of systems are generally referred to as the Clinical Decision Support System (CDSS) [16]. These types of systems generally focus on generating recommendations using artificial intelligence (AI) and machine learning techniques. However, they lack the ability to discover, link, and provide an analytical view of the process of clinical trials [2] under investigation. These trials may still be under investigation and not yet complete. On the other hand, this research work leaves the part of analysis for a specific disease, diagnosis, treatment, etc. to the user of this solution and focuses on presenting a unified system where data from a variety of sources can be obtained at one place and be able to perform any kind of analysis such as machine learning, data preparation tasks, profiling, and recommendations [13] as listed in the contributions and uniqueness section of this paper. This research does provide a real-time and robust view of the processes to support fast and reliable clinical investigation while the data are not yet complete or an investigation has been completed.

### 2.4. Comparison of the Proposed Solution to State-of-the-Art

The proposed framework differs from and supersedes the existing state-of-the-art across several perspectives: Firstly, existing approaches either focus on data standardization for interoperability and exchange of clinical data specifically or act on the data in silos. Secondly, most of the analytical frameworks’ work is only with specialized datasets, such as clinical trials only, electronic health records only, or sensory data only. Thirdly, all the above-discussed systems do not capture the building timeline of events (changes) and mostly work with static data by loading periodic batches. The proposed framework in this paper addresses these problems by: fusing data from various sources; building profiles for entities; maintaining changes in events over time for the entities of interest; and avoiding silos of analysis and computation. Moreover, the solution is designed to support streaming, batch, and static data.

## 3. Methodology

In this section, the terms and symbols used throughout this paper will be introduced first as preliminaries, and then the overall architecture of the framework will be presented.

### 3.1. Preliminaries

The terms used in this paper are of two types: those that describe entities or subjects for which a dataset is produced by a data provider, and those that describe or represent a process that relates to the steps a dataset undergoes. A process may involve subjects as its input or output, but vice versa is not possible.

- Terms
  ○ Entity: An entity E is a clinical concept, disease, treatment, or a human being related to data that can be collected, correlated to, and analyzed in combination with other entities. For example, a vaccine for COVID under investigation is considered an entity. Humans under monitoring for a vaccine trial comprise an entity. Clinical concepts, diseases, treatments, devices, etc. are the types of entities that will be referred to as “entity being investigated,” whereas human beings on which the entities are observed are referred to as “entity being observed”.
  ○ Subject: A subject S is an entity, i.e., SE for which a data item or a measurement is recorded. For example, a person is a human entity, and a vaccine is a clinical concept entity.
  ○ Subject types: A subject type is a subject for which data items can be recorded and can either be an entity being investigated or an entity being observed.
  ○ Domain: A domain D is a contextual entity and can be combined with a specific “entity being investigated” subject-type. For example, “breast cancer” is a domain, and viral drug is a domain. Moreover, in clinical terms, each high-level concept in Mesh ontology [17] is a domain. Similarly, “electronic health record” (also called EHR) is a domain.
  ○ Sub-domain: Just like a subject with sub-types, a domain has sub-domains, e.g., “cancer” is a domain and “breast cancer” is a sub-domain. Furthermore, sub-domains can have further sub-domains.
  ○ Schema: S The template in which measurements or real values of a subject are recorded is a schema. A schema specifies the types, names, hierarchy, and arity of values in a measurement or a record. Schema is also called type level records.
  ○ Instance: An instance I is an actual record or a measurement that corresponds to a schema for the subjects of a particular domain. An instance of an EHR record belongs to a subject “person” of entity “human,” with entity type as an “entity being observed”. Similarly, a clinical trial record for “breast cancer” investigation with all its essential data (as described in coming sections) is an instance of entity “trial” in the domain “cancer” with sub-domain “breast cancer” and is an entity of type “entity being investigated”. Moreover, a set {I} of instances is referred to by a dataset D such that D has a schema S.
  ○ Stakeholder: A person, an organization, or any other such entity that needs to either onboard their data in the framework for analysis, use the framework with existing data for insights, or do both is a stakeholder.
  ○ Scenario: A scenario S is a representation of a query that defines the parameters, domain, context, and scope of the intended use of the framework. For example, a possible scenario is when a stakeholder wants to visualize the timeline of investigations/trials for a certain “domain” (i.e., “breast cancer”) in the last two years by a particular investigation agency/organization. The scenario is then the encapsulation of all such parameters and context.
  ○ Use case: A use case UC is a practical scenario represented by steps and actions in a flow from the start of raw data until the point of analytical/processed data intended to show the result of the scenario.
  ○ Timeline: A timeline is normally the sequence of data-changing events in a particular entity being monitored. For example, in the case of a clinical trial, it is every change to any of its features, such as the number of registered patients, the addition/removal of investigation sites and the investigators, and/or the methods of investigation. These types of changing events need to be captured and linked with the timestamp they were captured on. These data (a type of time-series) are crucial for building time-series analysis of clinical entities and investigations. An example of time-series analysis may be determining the evolution of a vaccine over a certain time or between dates or determining the role of certain investigators with a particular background in a clinical trial over a certain period linked with the trials’ stages or phases.
- Processes
  ○ Data on-boarding: It is the process to identify and declare (if needed) the raw data schema S for a dataset D, identify and declare domain-specific terms (e.g., an ontology term), and declare limitations, risks, and use cases.
  ○ Data management: It is the process to bring the raw data under management that is already ‘on-boarded,’ as discussed above. It should declare and process data and type lineage [18] and be able to represent each data item with a timeline, hence treat every dataset as a time-series dataset. There is more on this in the following sections.
  ○ Data correlation: The framework is designed to on-board and manage the interrelated data coming from various resources. This process, which runs across all other processes, is meant to declare the correlations at each step to link technical terms to business/domain terms; for example, for a medical treatment for people with obesity, it should find all datasets that provide insights into these clinical terms. In each of the processes, this process is carried out at various levels with different semantics, as shown in the following sections.
  ○ Data analysis: This is the process to perform on-demand data processing based on pre-configured and orchestrated pipelines. When a scenario is provided, the pipeline is triggered, and various orchestrated queries are processed by the framework generating derived results that can be streamed/sent to the user and additionally stored in the framework (particularly in the lakehouse) for future use.
  ○ Reproducibility: It is a process to reproduce [19] the result of a scenario in case of failure, doubt, or correctness checking of the framework system.

### 3.2. Architecture

**Figure 1:**
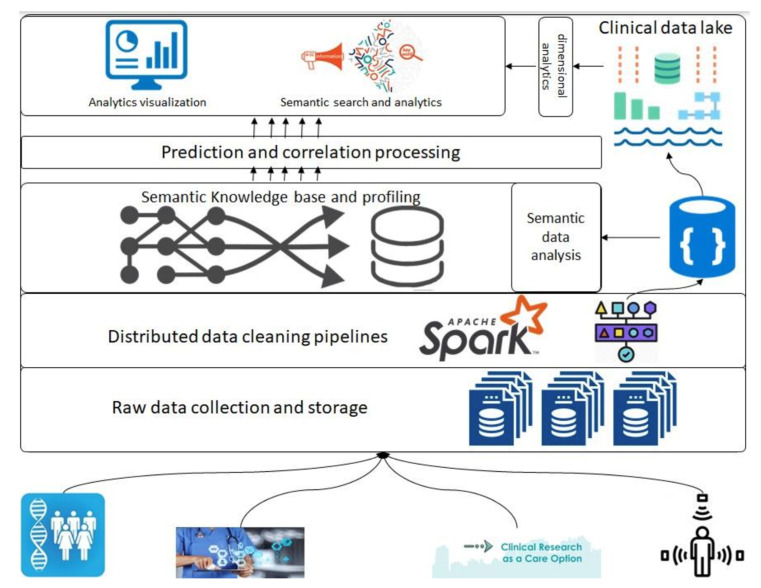
General architecture depicting different phases of data processing; analytics related to clinical data and publications.

In this section, we introduce the overall architecture of the framework. Figure 1 presents an abstract overview of the generic architecture with different components that will address the above-described objectives. A detailed flow of the architecture follows in the following paragraphs; however, firstly, a layer-wise functioning is presented in the framework. This architecture consists of various components, such as raw data collection at the bottom that acquires data that can be regularly scraped from data sources at the bottom. Next is the data-cleaning pipeline powered by Apache Spark; it is the component that is based on defined schemas and transforms all datasets to a unified data format (such as Parquet) before storing them in the data lakehouse. Next are the layers of semantic profiling and predictions. These layers are meant to process the data written to the data lakehouse and perform analytical tasks such as fusing data from various sources (academic clinical articles, clinical trials, etc.) and predicting profiles from clinical trial data. Finally, the application layer at the top represents dashboards and external applications requesting data from the bottom layers through some services.

Before any dataset is brought into the system, the raw data schema and any vocabularies or ontologies must be declared. This initial step is called data “on-boarding,” and it will become more visible in the following section when processes are discussed. After this on-boarding process, the data flows through the layers discussed above as follows: Storage of raw data to the data lake (right top in Figure 1), performing quality checking and mappings declared in the on-boarding process, and storing to the clinical data lakehouse. Clinical data lakehouse is also a file storage system with the ability to store each piece of data in a time-series format. For that purpose, we propose the usage of Hudi [20] which allows each piece of data written to the data lakehouse to be stamped/committed with a timestamp, thereby providing the ability to travel back in time. This data movement from the raw data to the data lakehouse is optional in the sense that the stakeholders who want to store ‘raw data’ can store it in the blob storage, whereas for others, the data can be directly brought to the data lakehouse. In either case, the framework can stream the data either from an external source or from the raw data storage. For the streaming of the data, we propose the use of “Kafka” along with akka-streaming technologies such as “cloudflow” [21].

Once the data are acquired and ingested into the data lakehouse, queries can be performed to do analysis and perform data correlation between various data sources. The analysis and correlation can be carried out on both static and inflight data, i.e., data ingested in a streaming fashion and hence requires the ability to merge/fuse with other streams.

When the data are related to a particular “entity being investigated,” it requires the correlation of these data to the appropriate academic research articles so that various stakeholders can relate the investigation to the state of the art in academia. The correlation methods and techniques are out of the scope of this paper; however, we do want to emphasize that this correlation and the mapping are critical to this framework. The results of the processing layer (the middle layer in Figure 1) are stored back in the data lakehouse for re-usability and availability. The querying engine that can be employed for this purpose is Apache Spark [22].

Finally, the data from the data lakehouse and the results of semantic analysis are meant to be streamed to applications of various types by various stakeholders, thus requiring the contracts to be defined before making such service requests. We emphasize the contracts here since the contracts are key to managing data lineage and explainability, as detailed in the following sections, and hence it is a prerequisite for an explainable system to have control over the data being moved and have a clear understanding of at what time what data in what format is being moved.

The framework is designed to be multi-tenant, having the flexibility to provide services and processing capabilities for private and/or public data management services. Those kinds of differences are made possible through proper access controls.

### 3.3. Processes and Methods

In this section, first an overview of clinical data sources will be presented, then some specific processes and methods (used interchangeably) that acquire, manage, fuse, and process these data sources to produce quality analytics will be presented. Next, all the components of the architecture that include data analysis modes servicing data to external analytical applications are presented, and finally, the concepts of lineage and reproducibility are presented since these concepts are at the core of the FAIR principal.

In the following subsections, each service is part of a layer in Figure 1. The layers in Figure 1 are abstract and hence overlook these services. In each process explained below, the respective layer of the architecture in Figure 1 is referred to.

#### 3.3.1. Data Sources

As visible from the framework, this framework is designed to on-board data that are related to real entities. These include various sources.

Public sources: First, there were data from clinical trial investigations conducted by public agencies that were published in their publicly available repositories. In this case, the data can be on-boarded by the published (as the stakeholder) to the framework so that it is not just available to the public (because it already is in the form of a public repository), but it is also available and meaningful because this framework makes the data integrated with other sources and provides an intelligent and smart overview and analysis. The clinical trial data mostly contain a wider range of information related to the sites of investigation, the investigators, their affiliations, treatments, and outcomes, among others. See [23,24] for a detailed overview of what a trial can contain. Moreover, publicly standardized clinical ontologies are also one of the key sources of data that would be on-boarded and used in multiple phases; they are used in the on-boarding process to map the raw data to clinical entities and concepts and map the clinical trials, investigators, and sites to clinical concepts.

Private sources: A second source of the data is a private agency that would like to analyze its own clinical investigation in the same domain as public data providers, hence on-board data from its private repository and get the analysis results in the examples of use cases and scenarios as described in sections below. Note that this framework is multi-tenant with proper access control mechanisms; therefore, the on-boarding of private data can be “timed” (delete or archive after a certain time limit).

Personnel/Electronic health records: These are data that can be on-boarded from private and public data providers. However, these data might include sensitive and personal information about valid and real human beings, who can be reluctant to share their data for privacy reasons. In that case, proper consent must be obtained before data are on-boarded, and the data must be anonymized using known anonymization techniques in the industry [25].

Academic Data: “Entities being observed” are not only appearing in the data from clinical trials as discussed above, but also occur as the latest research in these domains is published in the form of academic journals and conferences such as biomedical engineering and other journals. We consider these datasets (journals and conferences) as one of the key datasets that are publicly available and brought to the framework in increments and updates. As explained in the following sections, these datasets are correlated with clinical concepts in ontologies such as Mesh and others and with clinical trials. The usefulness of these datasets and their integration is of the utmost importance for clinicians and the pharmaceutical industry to correlate their findings with state-of-the-art academic advances.

Derived datasets: These are the datasets that result when the data are managed and correlation is performed and/or when there are updates and it is needed to pre-compute some results so that these results can be provided when requested without computing them on the fly, for obvious reasons such as the complexity of the analysis/query and the latency it takes to compute those results. Examples of these include correlating academic journals to clinical concepts, building trials and other timelines, generating time-series of certain data, and resolving entities to concepts, among others. In other words, these derived datasets could result in both semantic data (stored in ontologies) and non-semantic data stored according to a particular schema S. Since it is only allowed for a dataset to be persisted in the data lakehouse if and only if it has a schema, therefore each such derived dataset must be declared, annotated, and searchable. These are described in the following section of the ‘on-boarding’ datasets, and on-boarding can be for both internal and external datasets.

#### 3.3.2. Data on-Boarding and Discovery Process

**Figure 2:**
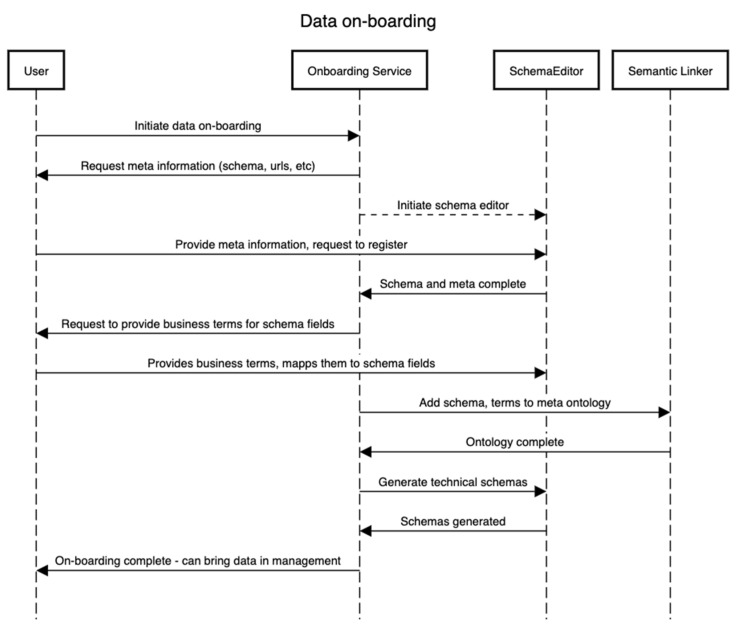
Data on-boarding activity sequence.

**Figure 3:**
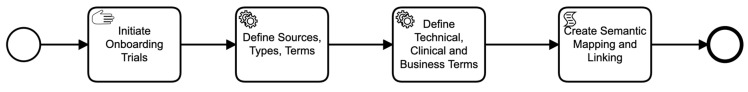
Data asset on-boarding business process.

This subsection details how the on-boarding service is designed to operate. This service is external to the framework as a separate entity since it needs to be independent of whatever process the framework follows. It is critical to the whole framework as this is the first point of entry for stakeholders that will likely be storing the data and requires knowledge about the domain of the data. Figure 2 explains this with an example. The data on-boarding process typically involves four entities/components. The sequence of steps presented in Figure 2 is complemented by the business process presented in Figure 3. We assume one wants to on-board a dataset D that contains or will contain the set {I} of the instance of a domain D for an “entity being investigated”. In that case, to identify the meaning of each field in D, e.g., if a field/variable has a certain value, it is needed/required to identify the type, domain, and its business or conceptual meaning (as shown in Figure 3). Therefore, in this process, defining both the technical schema S of the D as well as a conceptual schema are required. The technical schema is used during the data acquisition and processing, whereas the conceptual schema is used to construct the semantic meaning of the assets, such as for discoverability of datasets by other users.

**Figure 4:**
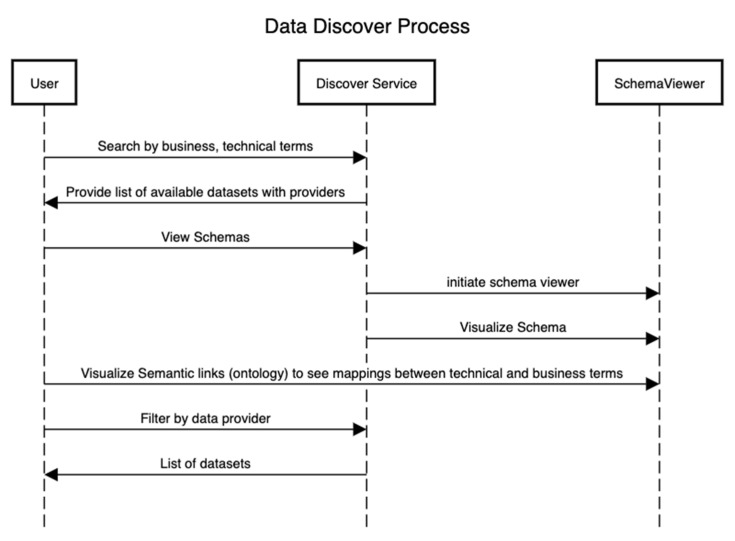
Data asset discovery process.

Figure 4 presents the sequence of steps that showcase the discovery of assets after the technical and conceptual schemas in Figure 2 and Figure 3 have been defined. In this process, an external user can be interested in using a particular dataset for the training of machine learning algorithms or other analytical algorithms if the data provider has consented to it.

#### 3.3.3. Data Management Process

**Figure 5:**
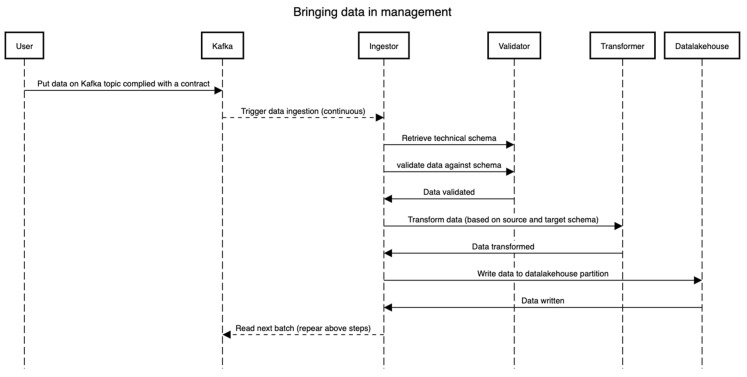
Data collection and transformation sequence.

The data management process cannot be instantiated prior to or in parallel with the on-boarding process. This is intended to make sure that each dataset that lands in the management is queryable, understood well by the framework, and of use to the stakeholders. This process relates to the two bottom layers in Figure 1. Once a dataset *D* is on-boarded (See the Section 3.3.2), the technical schema *S* is version controlled (we suggest using Git VCS for this) and stored in the schema repository. Figure 5 presents the flow of events, essentially depicting the pipeline through which the data flow (both static and dynamic). The technical schema *S* is first used by validating that the raw dataset (read from Kafka) *D* conforms to the schema *S*. Then, the Ingestor component performs necessary transformations such as validation on certain fields, extracting information from file names, etc. Then, the validator validates real data values against the schema. Lastly, the transformer components perform the transformation to match the table in the data lakehouse, wherein all types and formats of data are stored irrespective of their formats. In all these steps, the distributed framework Spark is leveraged, and all the steps can be traced using the lineage tracking APIs. Note that the transformer supports additional mappings (format changes/updates, time-zone information, among others) and creates a schema *S* for the data under management (i.e., in the data lakehouse). However, it is strictly monitored that the schema *S’* does not alter the meaning, names, and bounds set in the initial technical schema S in the on-boarding process. As visible in the diagram, the data can be streamed directly from an external source through the means of a streaming framework such as Apache Kafka [26], Akka streams [27], or any other such framework. The goal here is not to impose any technology but rather the capabilities of lineage and tracking. Note that by having a proper schema and the conformance of the data to that schema, one can trace back from the end to the start of the whole process to see where the data comes from and how it comes.

#### 3.3.4. Data Correlation

Data correlation (layer semantic linking in Figure 1) is about mapping data from various sources to one another such that it can easily be queried for understanding and mapping. As an example, when data for entities being observed in some domain (i.e., cancer) are on-boarded, this will include updates related to investigators, sites, treatments, medication, and outcomes. The first step is to map the personnel data (if available) from health records to the clinical trial investigation study. Moreover, another aspect is that each update to a dataset or to a clinical trial can contain a reference to a single instance of a person or investigation site, say an investigator, but with changes in the name or the spelling, among others. Similar is the case with sites, diseases, and medical conditions (components of clinical trial data). In all such cases, the mapping of all such individual occurrences from the data to real records in the system is carried out, and the ontology has to be built.

Moreover, academic data include references to clinical concepts (entities being observed) and sub-types and domains. Whenever an academic dataset is brought to be managed, the data points are correlated with the “data correlation” component to perform semantic search and produce records. These kinds of semantic results are useful for clinicians and pharmaceutical agencies to relate their outcomes to academic investigations. Therefore, data correlation is a key process that takes place each time there is an update or a new dataset in the system. Note that the clinical terms can also be referenced from clinical trials, and in fact, each clinical trial always relates to at least one clinical term, so the mapping must occur at the time of ingestion of each dataset. For this purpose, *semantic* mappers can be developed that will use clustering and text-based techniques to map incoming updates to certain entities to existing entities in the system, e.g., mapping updated clinical sites to existing sites, investigators to existing investigators, etc. Note that the clinical data available in public repositories or with private owners do not necessarily contain unique identifiers for these entities, and if they do, it is not synchronized across various repositories and data providers.

#### 3.3.5. Data Analysis

This subsection describes two types of analyses, and those can always be extended to other types. This process is part of the layers of semantic linking and correlation processing in Figure 1.

Offline/Periodic: As a first type of analysis, the vision is that data analyses that are required to be performed on updates to data or periodically. These include analyses that are heavy with high latency or analyses that are potentially going to be requested frequently in scenarios by various stakeholders/user of the framework. As an example, building the timeline of a clinical trial is performed each time there are updates to the datasets for that trial. Similarly, building timelines for investigators, treatments, and sites is also offline but actively performed when data arrive in the system. These kinds of datasets are referred to as projections or derived datasets, as described in the data sources section.

Moreover, another example of such analysis is building a feature store of clinical trial results; e.g., when an outcome of a trial is obtained, a feature is made out of it so that it can be used as input for statistical learning in machine learning for predictions and for further analysis.

#### 3.3.6. Data Services

The architecture is presented in Figure 1. Data services serve two types of data: (1) data about data (i.e., metadata) and (2) data itself. In the case of metadata, the framework offers the ability to search and discover what types of data exist in the system–e.g., a data user may want to know what data providers provide the data, which clinical trial repositories’ data exist in the system, what are the schemas of these data, how to obtain the data, and so on. As mentioned earlier, for this purpose, datahub [28] is one of the best examples. This service is closely coupled with a data on-boarding service; once data are on-boarded, they can be searched as explained above. For this kind of service, we propose using an external system called datahub, which is mature enough to perform this function. The separation is mainly because metadata is a slowly changing dimension of the data. Hence, it does not require streaming capabilities now, and redoing it is not wise either. Secondly, when the data are in the system, the framework offers the ability to query and stream the data to the user/end system. This service complements the metadata service since this cannot stream data that cannot be discovered by the former. Here, we insist on streaming services since, on the one hand, streams can be generalized to streaming larger chunks to support non-streaming application endpoints and streaming data to support transporting large datasets continuously and in mini chunks. The second service of serving data from the platform is available for each layer in Figure 1 since all the data end up in the data lakehouse.

#### 3.3.7. Lineage and Reproducibility

The ability to reproduce results retrieved from certain data using a set of algorithms and techniques is a key requirement for explainable and trustable systems. To do so, the framework supports techniques and methods to keep track of data lineage at the meta level, and then the framework is designed in such a way that, if needed, one can reproduce the expected result from the same data using the same algorithms. At the meta-data level, for each change (update) in the dataset that is being ingested into the system, if there is a certain modification (technical modifications such as a schema change), it is required to create a schema and deal with it internally as a separate dataset–as earlier described as the derived dataset or projected dataset. In this way, one can track the lineage at each point of each computation or calculation, and all these schemas for these datasets are available in the meta-data discovery service. Moreover, ontology is built of related concepts with the system to make sure that if an entity is related to, for example, a trial, then it can be tracked. Secondly, the data can be queried using the scenarios as explained below; these scenarios in fact define the various parameters such as time window and dataset, among others. With these parameters, one can always reproduce an expected result from a certain point in the pipeline since the windows and the scenarios are recorded.

### 3.4. Use Cases and Scenario

The framework supports a variety of use cases (UC), and we will explain some of the preliminary and easily understandable ones in this section. We relate use cases to stakeholders (users of the framework), and hence the design of the framework is intended to be specific and address the objectives. Moreover, each use case is assumed to include some steps, involving multiple parts of the framework in a certain order. In other words, a use case is related to a business process. Therefore, with each use case, we also show a tentative business process diagram. This can also be interpreted as an orchestration scenario, where the steps in the process are orchestrated. An orchestration scenario is different than the scenario described above in the sense that the orchestration scenario is about the steps of a process, whereas a general scenario is analogous to defining the scope of what data to retrieve as described above.

#### 3.4.1. Use Cases

- UC_1_: As a data provider, I want to bring the data into the framework so that they are available to other stakeholders for research and investigation.
- In this use case, a potential stakeholder can be a government organization that wishes to make a dataset D that it owns for a domain D and/or continuously performs investigations related to a particular “entity being investigated”. Therefore, with the help of the “on-boarding” process, the stake-holder along with the technical support should be able to declare related “concepts” domain terms, define a technical schema S for D, a conceptual schema S’, and provide an API (application programming interface) to access the data by the framework or place it in a raw data storage (again making it accessible by the framework).
- UC_2_: As a data provider, I want to bring the data into the framework so that I can use them in combination with other publicly available datasets, but I want my dataset to be private.
- In this case, the process is similar to above; however, this time, the data are private (tenant support) and can be queried through the service layer in combination with other publicly available datasets based on scenarios.
- UC_3_: As a stakeholder, I want to know the available datasets related to the domain breast cancer [28] and the timelines of trials in the last 3 years of investigation in this domain for the entities being investigated correlated with “entities being observed.”
- This particular use case deals with querying data that is correlated. The process starts by first using the on-boarding service to query existing available data using business/conceptual terms. This will enable the stakeholder to choose which datasets he/she is interested in. Then, a scenario is created based on the use case definition and forwarded to the service layer for execution. The service layer, with pre-executed correlation and mapping, answers the query in a streaming fashion.

#### 3.4.2. Scenarios

- S_1_: As a user (stakeholder), I want to get the set of instances {I} (data) for the last two months for the “entities being observed” E in the “viral disease” domain D with a sub-domain “COVID-19”.
- S_2_: As a user, I want a timeline (an analysis) of the entities E being observed in the “breast cancer” sub-domain for data providers from the Americas (or for a specific clinical trial data provider).
- S_3_: I want to get the analysis of successful trials in the last two years in the “intestinal disease” domain.
- S_4_: I want to get all the instances {I} for all the entities {E} in the “cancer” domain D with trial outcomes (see [23,24] for details on outcomes) to train a machine learning algorithm.
- S_5_: As a pharmaceutical company analyst (stakeholder), I want to see the duration of affiliation of all investigators from sites in Europe associated with clinical trials in the domain of “cancer”.

Online: These are the analyses, the scenarios (see examples in the following “Scenarios” section), that will be executed by the “data service” and might result in two options: (1) when the analysis (query) completes, stream/send the result back to the requested; (2) store the result in the data lakehouse and stream/send the result back. The second case needs to be implemented such that one can cache the analysis result in memory; if the request is repeated after a certain threshold (or another such metric can be defined), one can persist the analysis result to the data lakehouse.

All such analysis results at the time of deciding to persist to the data lakehouse will first go through the process of on-boarding. As described in the on-boarding process, each dataset that lands in the data lakehouse must have a schema S. In this case, a schema is registered, semantically annotated, and then the dataset is written to the data lakehouse for reasons described in the framework description and the ‘derived’ data sources description.

**Figure 6:**
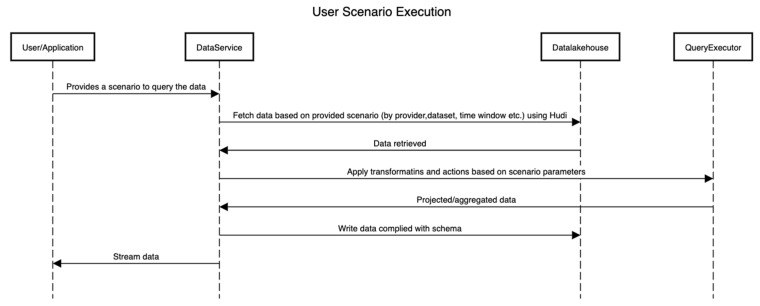
Sequence of activities in a scenario.

Figure 6 shows the sequence of activities that are performed to execute a scenario by interacting with the platform. Here, the internals of the platform are hidden, and only the interactions between components are shown.

**Figure 7:**
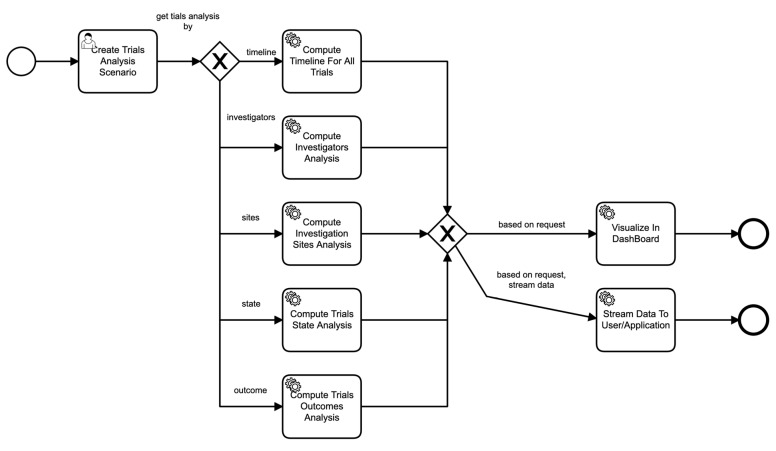
Business process of scenario execution.

Similarly, Figure 7 shows the process of a scenario based on parameters provided in the scenario. For example, if the scenario asks for the analysis of sites of clinical trials, then the data service invokes those particular components to perform those functions seamlessly, and so on.

## 4. Experiments, Results and Evaluation

This section presents implementation details, followed by results and the evaluation of results and computational advantages.

### 4.1. Implementation

A prototype version of the proposed framework is implemented with each component, as presented in Figure 1, to reflect the use cases and provide a minimal viable prototype (MVP). More specifically, we took the case of managing “clinical” trials as a case study for this prototype. Clinical trial data are obtained from the aforementioned repositories available publicly, where one can obtain a history of trials as well. The specific clinical trial repositories used are BSMO trials and CityGov trials, and for academic research manuscripts, we used research articles from well-known research journals such as “Journal of Clinical Investigation” [29] and Sage Journals [30]. We used Apache Hudi as the data storage format (using parquet file format), which is compact, provides time-series data checkpointed by any update/upserts, and provides efficient query processing using the query engine Spark. Apache Hudi was used since it supports parquet, which is format-agnostic, succinct, and fast.

For storage of data, Azure blob storage is used as the clinical datalake, where the raw data are stored, and it has also been used to store the ingested data in the Hudi format as Hudi tables (which are time-series and checkpointed), which is also called the data lakehouse. This storage selection is purely optional, and it can be any file system storage. Scala is used as a programming language, with Apache Kafka as a message-passing and data streaming framework and Apache Spark as a distributed data processing framework. Moreover, Lightbend cloudflow is integrated with these technologies and hence used in the replacement of microservices.

The process of data acquisition, management, and processing takes place as follows: First, we scrape data from available public repositories provided by providers and log it to two places: Kafka messages and datalake. Then, we ingest the data from Kafka (each Kafka message conforms to a schema defined in the Avro format specific for that dataset) in a streaming pipeline integrating Kafka and write to the data lakehouse (bottom layer in Figure 1). This step brings any format of data into a single format, and we can use our processing framework Spark with ease to perform any type of analytics (next layer from bottom in Figure 1).

We implemented a data analysis service (3rd layer from bottom in Figure 1) to correlate information from profiles, clinical trials, and trial subjects to identify the clinical profiles of people. This included various forms of information extraction and knowledge creation, such as the creation of the ontology to construct the relationship between these various datasets. Similarly, we also implemented a front-end to register assets (datasets and providers) so that we could later query and prove that such a concept does exist. We leveraged the ‘datahub’s data model [28] for that purpose and found that it is one of the best metadata models for registering and managing assets linked with glossary terms and business vocabularies as well as technical vocabularies and defining roles and ownerships.

Moreover, the clinical trial data contain textual data wherein personnel and investigation site profiles repeat. This type of data needs correlational and contextual analysis to deduce the real person from it. That is mainly because the person profiles are manually filled in by various people in the trial data or because the people are active in various sites and organizations, and hence a single profile can have a lot of variations. For example, a person’s name “Kasper Sukre” can be written as “K. Sukre”, “Kasper S”, “K. S”, etc. We therefore needed to resolve all those names to a single person entity. This entity is essentially a person entity in the relational schema of the clinical trial data that we take as a case study.

### 4.2. Results and Evaluation

We implemented textual person profile prediction using clustering algorithms (2nd layer from the top in Figure 1). We compare the results of two clustering algorithms with two different types of distance algorithms. In the first distance algorithm, since a person profile contains various features such as name, address, cell, email, association, and similarly, address and association can have further sub-fields; therefore, we compute the edit distance between each sub-field separately, which is a nested distance. Finally, we take an average of all distances. In the second distance algorithm, we apply a generic edit distance to the whole person profile as a string and then apply the clustering algorithms. Table 1 shows the results of the two algorithms for the two variants separately.

As can be seen in Table 1, the algorithm EM consistently performs better than K-Means on both variants of the distance algorithm. Since the algorithms are not 100% accurate, and it makes sense, we therefore employ a quality check on the results to verify the final person recommendation that is also required since we cannot completely rely on algorithms for personnel data.

Next, we must also indicate that we were able to stream data from the scraper that scrapes the data from various repositories simultaneously in parallel and were able to write to the same table (the Apache Hudi table) time-series data at around 5 times faster than normal upserts to a relational database. Since the framework is designed to poll data sources regularly and as soon as data are available, it is seamlessly brought into the system using the automated pipeline, and the algorithms to link and personalize are performed on the fly on the incoming data. Note here that, when any new data arrives in the system, the algorithms implemented above (analytical and predictive) are also part of the pipeline orchestration. Therefore, this avoids any cron jobs, periodic, and/or manual jobs to perform these tasks, and if necessary, data can already be streamed or made available to applications such as Kafka messages. This kind of automation, fusion, and parallelism makes this framework unique in that it speeds up the manual process used by the existing systems that follow the process of scraping data, ingesting it periodically, and then running cron jobs to perform analytics. Apart from the automation of the process, existing systems, as explained in the state-of-the-art, also lack data fusion of trial data, academic articles, Medical Subject Heading ontologies, and linking profiles.

## 5. Discussion

In this paper, we present a healthcare framework that enables us to handle health-related data. When clinical trials are under investigation and need an advanced and automated system to speed up the trial process and monitor the treatments, investigations at sites, their outcomes, and other related aspects of trial management, we presented a framework that streams data from various sources, fuses the data semantically, applies algorithms to resolve duplicates, and handles the history of events. This does not only speed up the trial analysis process compared to the existing methods; it also enriches the trial analysis and gives a complete context with the latest research from academia, semantic ontologies, and a holistic view of events. The framework is designed to support the declaration of schemas (type-level information) and metadata related to various data that comes into the framework. This enables exploring any analysis being made over the data and making it accessible to third parties as well as government and other agencies to use data from data providers, express interests, and/or bring their own data to the system to be used by other such users of the system.

We have presented scenarios in which this framework can be utilized. Not limited to these scenarios, the framework can be extended to support a variety of specialized data systems; for example, they include complex event processing, weather monitoring, and traffic and air management, among others. This is all because we have leveraged the data lakehouse technology and provided abstractions that can be used for any kind of querying of the data. Moreover, the framework is designed to support data lineage, which helps in achieving data accuracy and provenance. This is mainly obtained by keeping track of each transformation and operation performed within the framework, and thus it can be reproduced either at the operation level, data level, or type level. All these help in evidence-based decision-making and thus prove that the framework conforms to and fulfills the critical needs of a system needed for healthcare-related sensitive data.

Furthermore, the framework is designed to be extensible, and it envisions the inclusion of automated feature engineering required to generate recommendations from historical data based on statistical models and methods. This can further be used to plug and play models in the framework, which leads to using data from one model provided by a model provider or a third party using the model and data from separate users. Hence, this framework in this paper is limited to clinical data processing, lineage tracking, and governance and applies and extends to other areas of research as well.

## 6. Conclusions

In this research article, we presented a detailed overview of the need for a framework for clinical investigation to speed up the process of clinical trials for medical equipment, vaccines, and other such products, especially in the event of pandemics such as COVID-19 [4,31]. This is necessary since a delay in such matters causes the death of humans, and saving a single life is analogous to saving humanity. Moreover, the presented framework has the potential to provide evidence-based data analysis through lineage tracking; data governance capability to explore, visualize semantics and links, and possibly make decisions among various data sets available in the framework for use; and process data at state and in motion. We have presented use cases that showcase the usage of the framework in different scenarios and how it can be used by both private, public, and private-public organizations. The framework is extendable, encapsulates the abilities to include other domains, provides support for semantic querying at the governance level (i.e., at the data assets and type assets level), and is designed towards a data and computation economic model. Finally, it supports evidence-based decision tracking, and hence nothing is lost or unknown in the process of evaluation and analysis, which follows the FAIR principle presented earlier in the introduction section. Future work includes a detailed investigation of the specifics of each part of the framework in the domains of semantics, learning models, and the incremental evaluation of queries that need to be evaluated on updates in real-time data processing.

## Figures and Tables

**Table 1:** The results of the two clustering algorithms for two different variants of distance algorithms.

	General Edit Distance	Feature-Wise Edit Distance
K-Means	79.43%	85.4%
EM	83.54%	88.9%

## Data Availability

Not applicable.
